# Multimodal imaging analysis of autosomal recessive Parkinson’s disease

**DOI:** 10.1007/s12149-025-02053-4

**Published:** 2025-04-24

**Authors:** Başak Soydaş-Turan, Gül Yalçın-Çakmaklı, Eser Lay Ergün, Hacer Dasgin, Kader Karli Oguz, Bulent Elibol, Omer Ugur, Bilge Volkan-Salanci

**Affiliations:** 1 Department of Nuclear Medicine, Hacettepe University School of Medicine, Ankara, Türkiye; 2 Department of Nuclear Medicine, Kastamonu Training and Research Hospital, Kastamonu, Türkiye; 3 Department of Neurology, Hacettepe University School of Medicine, Ankara, Türkiye; 4 Medical Imaging Techniques, Mudanya University Vocational School, Bursa, Türkiye; 5 National Magnetic Resonance Imaging Center (UMRAM), Bilkent University, Ankara, Türkiye; 6 Department of Radiology, Davis Medical Center, University of California, Sacramento, USA; 7 Institute of Neurological Sciences and Psychiatry, Hacettepe University, Ankara, Türkiye

**Keywords:** Parkinson’s disease, Monogenic, Multimodal imaging, 18F-DOPA, 18F-FDG

## Abstract

**Objective:**

Pathophysiological backgrounds of idiopathic Parkinson’s disease (IPD) and autosomal recessive monogenic Parkinson’s disease (AR-PD) have common features that can be assessed through multimodal imaging. In this study, the striatal and myocardial dopaminergic innervation, brain 18F-FDG metabolism, resting-state functional activity of basal ganglia network (BGN) and white-matter (WM) microstructure were evaluated in AR-PD with respect to IPD, to investigate whether AR-PD can be subtyped as “brain-first” parkinsonism according to recent etiopathogenetic classification effort.

**Methods:**

Forty patients (17 with Parkin, 3 with DJ-1 mutations and 20 with IPD) were included. Striatal dopaminergic innervation was assessed semi-quantitatively by 18F-DOPA PET, and cardiac 18F-DOPA uptake was also evaluated. Brain 18F-FDG PET images were evaluated visually. Resting-state functional MRI and diffusion tensor imaging (DTI) were used to assess the BGN activity and WM microstructural alterations.

**Results:**

AR-PD patients showed significantly decreased 18F-DOPA uptake in caudate corpus compared to both IPD and controls, with a more symmetrical striatal dopaminergic denervation. Myocardial 18F-DOPA uptake in AR-PD was similar to controls, while it was significantly reduced in IPD. There was no significant difference in cortical 18F-FDG metabolism and functional activity of BGN between PD groups. The DTI data revealed more extensive WM microstructural damage in AR-PD compared to IPD.

**Conclusions:**

AR-PD group showed additional significant decreased 18F-DOPA uptake in caudate corpus and more symmetrical striatal denervation. Additionally, relatively preserved myocardial innervation, cortical metabolic and WM microstructural changes suggest the possibility of “brain-first” type progression in AR-PD. Also, 18F-DOPA PET/CT may be a practical tool for evaluating dopaminergic innervation of striatum and heart together, but further evaluation is needed in this area.

**Supplementary Information:**

The online version contains supplementary material available at 10.1007/s12149-025-02053-4.

## Introduction

Parkinson’s disease (PD), characterized by nigrostriatal dopaminergic denervation, is clinically and genetically heterogeneous. The diagnosis requires the presence of bradykinesia, resting tremor and/or rigidity, even though patients also exhibit a wide range of non-motor symptoms (NMS), such as disturbances in cognition, sleep, olfaction and autonomic nervous functions, even prior to the onset of motor symptoms [1]. Lewy pathology (LP) is the hallmark of the illness, which has been identified in dopaminergic neurons in substantia nigra (SN) pars compacta, explaining the classical motor symptoms of PD. LP mainly composed of pathological alpha-synuclein (a-syn) accumulation, which has subsequently been identified throughout many brain regions, corresponding to all motor and NMS [1]. Recently, it has been suggested that idiopathic PD (IPD) can be subtyped as “body-first” or “brain-first” depending on the clinical features and spread of a-syn pathology. In the “body-first” subtype, pathology starts predominantly in lower brainstem, thought to result by spreading of a-syn pathology from the gastrointestinal and cardiac autonomic nerve terminals, and followed by caudo-rostral propagation in brain. In contrast, in the “brain-first” subtype, limbic-amygdala system is the predominant site of initiation for pathologic a-syn accumulation, eventually spreading in rostro-caudal direction with earlier and faster involvement of SN [2]. Supported by multimodal imaging studies [3, 4], these hypothetical subtypes present a pathophysiological frame to understand and foresee the prognostic differences in IPD.

On the other hand, there are also numerous monogenic forms of PD, usually indistinguishable from IPD on clinical background. However, some of the monogenic forms differ from IPD by showing no or conspicuous a-syn pathology [5], suggesting that phenotypic differences based on the development and progression pattern of LP may not be valid for these genetic forms. This is particularly true for the commonly seen autosomal recessive monogenic PD (AR-PD) forms caused by Parkin, PINK1 and DJ-1 mutations. Since the NMS such as constipation, REM sleep behavior disorder (RBD) and hyposmia are rare at the onset and develop relatively late, it has been suggested that this group of AR-PD may phenotypically fit the “brain-first” subtype of IPD [4] and might share similar prognostic milestones.

To explore the pathologic distribution and progression in vivo, previous studies have shown striatal-cardiac dopaminergic denervation, cortical metabolic and functional changes, and white-matter (WM) microstructural alterations in IPD [6–10]. For example, striatal dopaminergic deficit is typically most severe in the posterior putamen [6]. Myocardial uptake of radiolabeled neurotransmitters is generally reduced in IPD [7]. Using brain 18 F-FDG PET/CT, the PD-related pattern has been repeatedly validated [8]. Disease-related resting-state functional magnetic resonance imaging (rs-fMRI) changes are defined according to motor and NMS [9]. An increasing number of diffusion tensor imaging (DTI) studies have demonstrated that alterations in fractional anisotropy (FA) value, one of the most commonly used DTI parameters, in specific brain regions can effectively differentiate PD patients from healthy controls as well as atypical Parkinsonian syndromes, suggesting that FA could serve as an imaging biomarker [10]. However, there is limited data about these changes in AR-PD. In this regard, a multimodal imaging may provide valuable insights for understanding the pathophysiological grounds of AR-PD. For this purpose, we comparatively analyzed the combined data of 18 F-DOPA and 18 F-FDG PET/CT, rs-fMRI, DTI in AR-PD and IPD patients, and investigated whether AR-PD can be subtyped as “brain-first” with multimodal imaging clues.

## Materials and methods

### Subjects and clinical assessment

Forty patients (17 with Parkin and 3 with DJ-1 mutations, 20 IPD shown to have no known PD mutations) with a diagnosis of PD based on the UK Brain Bank criteria [11] participated in the study. PD patients were prospectively recruited from the neurology clinic at Hacettepe University Hospitals between July 2021 and September 2022. Genetic analysis was performed in collaboration with Department of Medical Biology and Genetics [12] and in ROPAD study [13]. Since the phenotype associated with DJ-1 mutations is generally indistinguishable from that of patients with Parkin mutations and because both mutations are associated with mitochondrial dysfunction and oxidative stress, and due to their rarity, they were evaluated together in the AR-PD group.

Age, gender, disease onset, clinical asymmetry, history of diabetes mellitus and heart failure were noted. Existence of NMS such as constipation, RBD and hyposmia were investigated by detailed clinical questionnaires with a special focus on the time of onset, whether they were present since the time of diagnosis or emerged recently. The severity of motor symptoms was assessed by the Movement Disorders Society Unified PD Rating Scale motor score (UPDRS-III) [14] and the stage of disease was determined using the modified Hoehn–Yahr scale while the patients were on medication.

The mini-mental state examination, Beck’s depression inventory and, a neuropsychological test battery including enhanced cued recall or verbal memory processes, trail making test A-B, Stroop test TBAG form, semantic (animals in 1 min) and phonemic (words beginning with “S” in 1 min) fluency, and clock drawing tests (4-point version) were performed. The administration and scoring of these tests were done according to previously described standards [15–18].

We compared 18 F-DOPA PET/CT images of PD patients with a control group consisting of age- and gender-matched patients who had undergone 18 F-DOPA PET/CT with a diagnosis of medullary thyroid cancer (*n* = 12) and insulinoma (*n* = 2), since we could not get approval from our local ethical committee for PET/CT scan on healthy individuals. Patients with known diabetes mellitus and/or heart failure were excluded from the cardiac innervation analysis. As a result, myocardial images of 10 patients and brain images of 13 patients were included in the 18 F-DOPA control group. MRI data of PD patients was compared with age- and gender-matched, right-hand dominant healthy volunteers (*n* = 9). None of the participants in the 18 F-DOPA and MRI control groups had a psychiatric/neurological disease.

This study was approved by the local institutional ethical board of Hacettepe University Faculty of Medicine (Approval Number: 2021/18–43). Written informed consent was obtained from all the participants and/or a family member before participating.

### Image acquisition and analysis

All patients were examined with 18 F-DOPA PET/CT. 18 F-FDG PET/CT and MRI could not be obtained in 1 AR-PD patient. Two AR-PD patients were excluded from the MRI group due to left-hand dominance. Seventeen IPD patients were evaluated by 18 F-FDG PET/CT, rs-fMRI and DTI. The MRI of the 15 IPD patients could be analyzed. All imaging was carried out in off-state dopaminergic medication (12-h period).

### PET/CT acquisition

PET/CT scans were acquired on a Discovery IQ PET/CT (GE Healthcare, Chicago, IL, USA). Both 18 F-DOPA and 18 F-FDG PET/CT were performed after at least 4 h fasting. All patients had a blood glucose level lower than 150 mg/dL before administration of 18 F-FDG.

185 MBq of 18 F-DOPA was administered intravenously for presynaptic dopaminergic imaging, while also aiming to assess cardiac 18 F-DOPA uptake. Since images taken between 30 and 90 min after injection are sufficient to reflect the intracellular uptake of 18 F-DOPA and its decarboxylation into 18 F-Fluorodopamine [19], patients were scanned for cardiac imaging after a 60-min uptake period (3 min/bed) and for brain imaging after 90 min (10 min/bed), following a low-dose CT for attenuation correction. For the control group, routine whole-body 18 F-DOPA PET/CT images obtained from the vertex to mid-thigh at 60 min after the injection (3 min/bed) were used for cardiac assessment. Additional brain images were taken after a 90-min uptake period.

Under standardized conditions (dimmed light-quiet room), 185 MBq of 18 F-FDG was injected intravenously on a different day. Sixty minutes after injection, brain 18 F-FDG PET images were acquired (10 min/bed) following a low-dose CT.

All PET data were reconstructed with an iterative algorithm. Images were evaluated on an AW4.6 (GE Healthcare).

### 18 F-DOPA PET/CT analysis

Brain images were semi-quantitatively evaluated to identify uptake in the striatum. A 10 mm spherical region of interests (ROIs) was drawn on the caudate head-corpus, anterior–posterior putamen, SN and occipital regions at both sides. The maximum standard uptake value (SUVmax) of these regions was noted to calculate the specific region-to-occipital ratio (SOR) which was defined as striatal/SN uptake minus ipsilateral occipital uptake, divided by occipital uptake. These values were calculated for each hemispheric region, and then left and right hemispheric data were averaged. Using the same formula, SOR was calculated for the ipsilateral and contralateral hemispheres which were determined by the initially affected hemibody and UPDRS-III.

Cardiac images were visually evaluated to identify uptake in the myocardium (normal/decreased). For semi-quantification, 15 mm spherical ROIs were drawn on the apex, septum, lateral wall of the left ventricle to calculate the SUVmax. The average of SUVmax in these three ROIs was used as the value for the whole myocardium. The background SUVmax was obtained by drawing a 15 mm spherical ROI on the ascending aorta. To assess myocardial 18 F-DOPA uptake, values obtained from left ventricle walls were proportioned to background [19].

### 18 F-FDG PET/CT analysis

Cortical–subcortical regions were graded visually as normal, hypermetabolism, mild, moderate or severe hypometabolism by the consensus reading of 3 nuclear medicine physicians blinded to the clinic. Cortex ID Suite 2.1 (GE Healthcare) which uses a standard template of ROIs for cortex to calculate mean counts and statistically compares these counts with the normal database following spatial normalization of PET data was used to assist interpretation.

### MRI acquisition

Images were acquired with a 3.0 T Signa Architect scanner (GE Healthcare) using a 48-channel head coil. Participants were instructed to lie still with their eyes closed, not to think of anything, not to fall asleep. Rs-fMRI were acquired using a gradient EPI sequence (TR/TE = 1000/25 ms, FOV = 192 mm, 164 volumes, voxel size = 4 mm^3^). DTI data were collected with the following settings: TR/TE = 4308/110 ms, 36 independent directions, maximum b = 1000 s/mm^2^, voxel size = 3 mm^3^. Anatomical imaging included sagittal 3D T1 W imaging applied MP-RAGE scan (TR/TE = 8.5/3.25 ms, FOV = 256 mm, 264 slices, voxel size = 0.6 mm^3^).

### Preprocessing-analysis of Rs-fMRI

All preprocessing steps were performed using SPM12 (https://www.fil.ion.ucl.ac.uk/spm/software/spm12/) and the Conn toolbox (https://www.nitrc.org/projects/conn) implemented in MATLAB2020b (The MathWorks, Natick, MA, USA). The first 4 images were discarded for each participant to ensure steady-state longitudinal magnetization. The default preprocessing steps including spatial realignment, motion correction, normalization to Montreal Neurological Institute (MNI, Montreal, QC, Canada) space, spatial smoothing with an 8 mm full-width-at-half-maximum 3D Gaussian filter were applied to co-registered structural and functional data.

Basal ganglia network (BGN) seed regions were segmented for each participant on T1 W images using the WFU Pick atlas (https://www.nitrc.org/projects/wfu_pickatlas/). Seed ROIs were defined bilaterally in the caudate, putamen, globus pallidus, subthalamic nucleus and SN. Seed-based connectivity analysis was applied to characterize the connectivity patterns within groups, as well as to identify activity differences between groups by performing *T*-tests. The threshold for significance was set to an uncorrected-*p* < 0.001 for the voxel level, family-wise error (FWE)-*p* < 0.05 for the cluster level and *k* > 80 voxels for the cluster size.

### Preprocessing-analysis of DTI

The diffusion data were corrected for susceptibility-induced geometric and eddy current distortions, head motion. Whole-brain WM skeleton-wise analysis was performed using tract-based spatial statistics (TBSS) [20] implemented in FMRIB Software Library v6 (FSL, Oxford, UK; http://www.fmrib.ox.ac.uk/fsl) to construct a single FA image from the WM skeleton for each participant. The FA images were obtained by fitting a tensor model to each voxel. The Brain Extraction Tool, as part of FSL, was used to remove non-brain voxels. Then, the FA images were registered into MNI space. A mean FA skeleton representing all major WM tracts was created by averaging the registered FA images.

Voxel-wise differences between PD groups were performed on the whole-brain mean FA skeleton to identify significantly affected WM tracts using the general linear model. The TBSS framework with non-parametric permutation analysis (5000 permutations) was used to control for multiple testing errors and FWEs. The threshold-free cluster enhancement option was used to avoid the selection of an arbitrary cluster-forming threshold. An FWE-*p* < 0.05 was considered significant. Results were shown in John Hopkins University WM tractography atlas within FSL.

### Statistical analysis

Statistical analyses were performed using SPSS v23.0 (IBM, Armonk, NY, USA) and *p* value < 0.05 was used for statistical significance. Data were presented as mean ± standard deviation and as median (min–max) for normally and nonnormally distributed continuous variables, respectively, and as the frequencies (percentages) for categorical variables. All continuous variables were tested for normal distribution using the Shapiro–Wilk test.

Normally or nonnormally distributed continuous variables from two independent groups were compared using the independent sample *T*-test or the Mann–Whitney *U* test, respectively. If the variables were categorical, the Pearson Chi-square test was used. Striatal-myocardial 18 F-DOPA uptake in AR-PD was compared to the same variables in IPD and controls using the one-way analysis of variance (ANOVA) with post-hoc LSD when the variables were normally distributed. If not, the Kruskal–Wallis test was applied with post-hoc Dunn’s test. For multiple comparisons, a Bonferroni correction was applied. Comparisons of striatal 18 F-DOPA uptake between hemispheres were made using a paired sample *T*-test for normal distribution and a Wilcoxon test for nonnormally distribution. Multivariable linear regression analysis was performed with the backwards elimination method to estimate the effect of various variables (age, gender, disease duration, UPDRS-III score and diagnostic group, either AR-PD or IPD) on 18 F-DOPA uptake in the caudate corpus and whole myocardium.

## Results

### Clinical characteristics and cognitive profiles

Patients’ characteristics and cognitive profiles are presented in Suppl. Table 1. The AR-PD group had younger patients (43 vs. 52 years, *p* = 0.003) with earlier disease onset (29 vs. 46 years, *p* < 0.001), longer disease duration (13 vs. 5 years, *p* < 0.001) and higher Hoehn–Yahr stage (2 vs. 1, *p* < 0.001) compared to IPD. Only presence of hyposmia differed statistically between AR-PD and IPD, which was more prevalent in IPD. The Z score of attention in the AR-PD group was poorer than IPD, while no differences were found in other cognitive domains between the groups.

### Striatal and myocardial 18 F-DOPA uptake

**Fig. 1:**
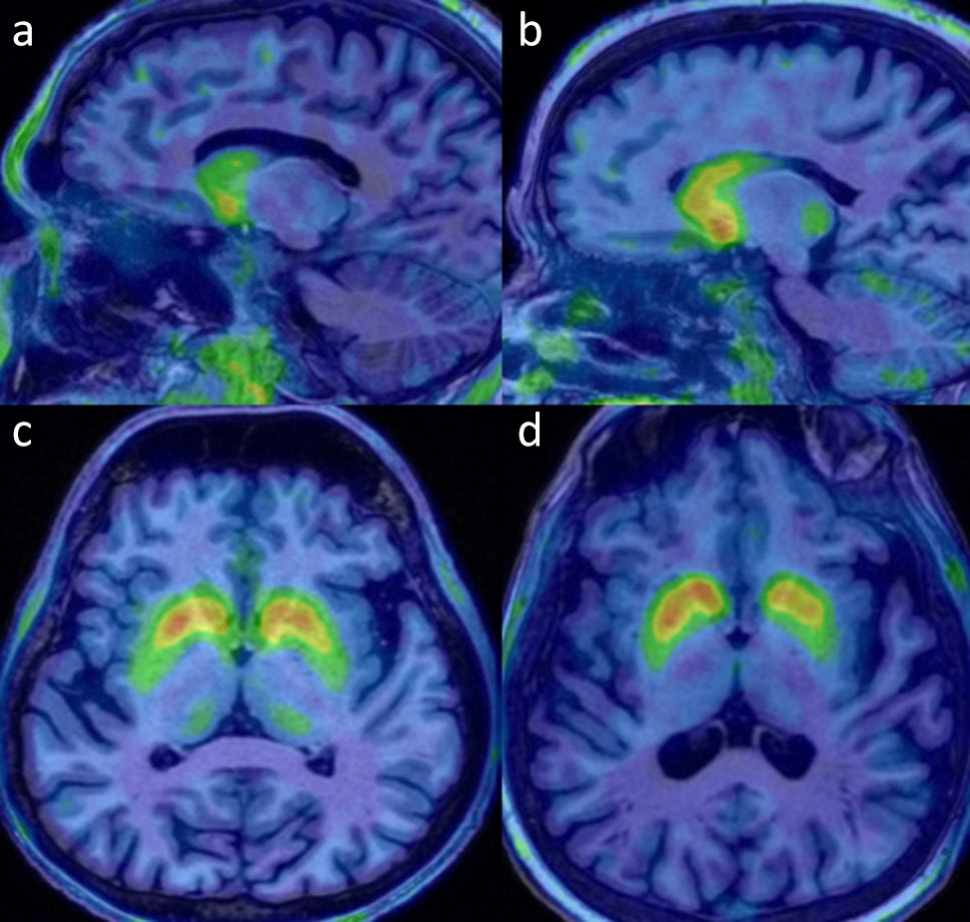
Striatal 18 F-DOPA PET/CT images. **a**, **b** 18 F-DOPA uptake in caudate was more decreased in AR-PD (**a**) compared to IPD (**b**) whose had same disease duration (13 years). **c**, **d** AR-PD patient (**c**)with right-side onset and 5-year disease duration showed symmetrically decreased 18 F-DOPA uptake in bilateral posterior putamen, whereas asymmetrically decreased 18 F-DOPA uptake was observed in contralateral putamen in IPD patient (**d**) with similar disease onset side and duration

A significant difference in 18 F-DOPA uptake was observed in the anterior and posterior putamen between PD patients and controls. In addition, the AR-PD group showed a significant decrease in 18 F-DOPA uptake in the caudate corpus compared to both IPD (Fig. 1a, b) and controls. Also, 18 F-DOPA uptake in SN was relatively decreased in AR-PD without reaching significance (Table 1).

**Table 1:** Comparison of 18 F-DOPA uptake in striatum, substantia nigra (specific region-to-occipital ratios) and myocardium

	Controls (*n* = 13)	AR-PD (*n* = 20)	IPD (*n* = 20)	*p*
Caudate head	2.09 ± 0.40	1.73 ± 0.45	1.88 ± 0.44	0.076*
Caudate corpus	2.12 ± 0.43	1.16 ± 0.54^a,c^	1.79 ± 0.45	< 0.001*
Anterior putamen	2.51 ± 0.46	1.43 ± 0.49^a^	1.61 ± 0.40^b^	< 0.001*
Posterior putamen	2.51 ± 0.51	0.80 ± 0.29^d^	0.88 ± 0.33^e^	< 0.001**
Substantia nigra	0.75 ± 0.30	0.61 ± 0.31	0.76 ± 0.19	0.177*

	Controls (*n* = 10)	AR-PD (*n* = 19)	IPD (*n* = 20)	*p*
Septum/mediastinum	1.83 ± 0.30	1.76 ± 0.26	1.54 ± 0.25^b,c^	0.010*
Lateral wall/mediastinum	1.62 ± 0.16	1.66 ± 0.26	1.37 ± 0.25^b,c^	0.001*
Apex/mediastinum	1.54 ± 0.26	1.51 ± 0.25	1.32 ± 0.18^c^	0.018*
Whole-myocardium/mediastinum	1.66 ± 0.23	1.64 ± 0.23	1.41 ± 0.21^b,c^	0.003*

*One-way ANOVA test. In post-hoc LSD analysis with a Bonferroni correction: ^a^*p* < 0.05 (AR-PD vs. controls), ^b^*p* < 0.05 (IPD vs. controls), ^c^*p* < 0.05 (AR-PD vs. IPD)

**Kruskal–Wallis test. In post-hoc Dunn’s test (pairwise comparisons with a Bonferroni correction): ^d^*p* < 0.05 (AR-PD vs. controls), ^e^*p* < 0.05 (IPD vs. controls)

The interhemispheric asymmetry of striatal 18 F-DOPA uptake was not significant in the AR-PD group, whereas 18 F-DOPA uptake in the contralateral caudate corpus, anterior and posterior putamen, and SN showed a greater reduction in IPD (Table 2, Fig. 1c, d).

**Table 2:** Comparison of 18 F-DOPA uptake values (specific region-to-occipital ratios) between hemispheres

	AR-PD (*n* = 20)			IPD (*n* = 20)		
	Ipsilateral	Contralateral	p	Ipsilateral	Contralateral	*p*
Caudate head	1.59 ± 0.54	1.73 ± 0.72	0.246	1.97 ± 0.51	1.78 ± 0.50	0.100
Caudate corpus	1.12 ± 0.65	1.08 ± 0.68	0.691	1.90 ± 0.44	**1.67 ± 0.56**	0.041*
Anterior putamen	1.36 ± 0.68	1.28 ± 0.35	0.510	1.73 ± 0.45	**1.48 ± 0.42**	0.005*
Posterior putamen	0.80 ± 0.45	0.77 ± 0.36	0.668	1.00 ± 0.47	**0.76 ± 0.30**	0.019*
Substantia nigra	0.58 ± 0.41	0.56 ± 0.31	0.774	0.84 ± 0.24	**0.69 ± 0.23**	0.034*

*Bold values represent statistically significant results

**Fig. 2:**
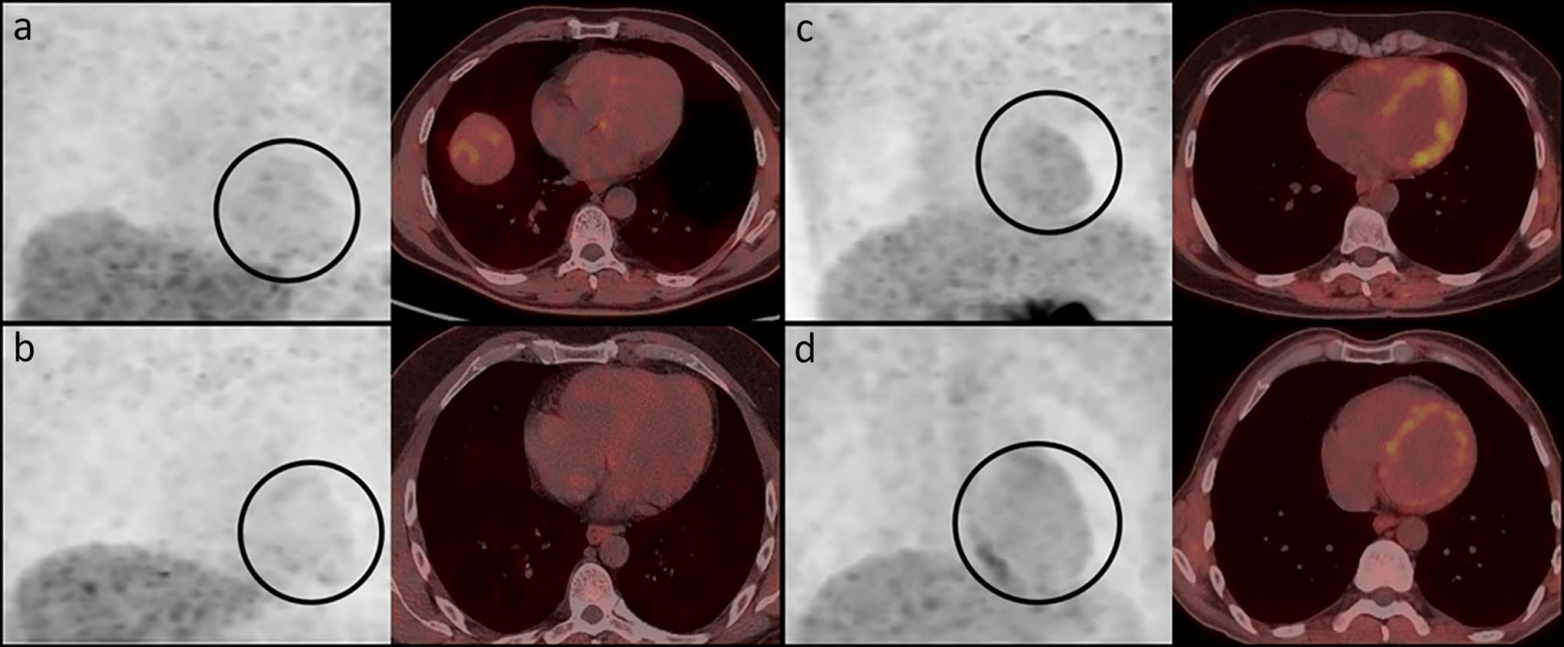
The maximum intensity projection (with the heart highlighted by a black circle) and trans-axial myocardial 18 F-DOPA PET/CT images of each patient are presented side by side. Decreased myocardial 18 F-DOPA uptake was observed in IPD patients with disease duration of 3 years (**a**) and 5 years (**b**), whereas relatively preserved myocardial 18 F-DOPA uptake was seen in AR-PD patients with 8 years (**c**) and 15 years (**d**) of duration

Visual analysis revealed that myocardial 18 F-DOPA uptake decreased in 5 AR-PD and 12 IPD patients (*p* = 0.034). Semi-quantitatively, myocardial 18 F-DOPA uptake in the AR-PD group was similar to controls, whereas it was significantly reduced in the IPD (Table 1, Fig. 2). Although it is not clear whether the disease status (medullary thyroid cancer and insulinoma) in our control group affects cardiac dopa decarboxylase activity, our data closely resemble the 18 F-DOPA cardiac findings in healthy individuals reported in the study by Goyal et al. [19].

When each PD group was evaluated separately, there was no significant difference in age, disease duration and clinical scores between patients with reduced and preserved myocardial 18 F-DOPA uptake (Suppl. Table 2). AR-PD patients with decreased 18 F-DOPA uptake in myocardium had a longer median disease duration (13 years) than IPD patients (5 years, *p* = 0.008).

Since the majority of our AR-PD group consisted of patients with Parkin mutations, our results mainly reflect this cohort. When the results of the DJ-1 patients were evaluated separately, similarly, we observed that the caudate corpus was the most affected region after the posterior putamen. There was no interhemispheric asymmetry, and myocardial 18 F-DOPA uptake was not reduced compared to controls (Suppl. Table 3). Therefore, pathophysiologically similar conditions may also be present in these patients. When DJ-1 patients were excluded, there was no change in our 18 F-DOPA findings, either (not shown).

Since the region that differed between the PD groups in the striatal structures was the caudate corpus, this area was evaluated in multivariable linear regression analysis, in addition to the myocardium. According to the results of this analysis, age, gender, disease duration and UPDRS-III score were removed from the model since they had no effect on caudate corpus and whole myocardial 18 F-DOPA uptake. AR-PD group was related to lower 18 F-DOPA uptake in the caudate corpus (*p* = 0.002, adjusted *R*^2^ = 0.203, Suppl. Table 4) and higher 18 F-DOPA uptake in the myocardium (*p* < 0.001, adjusted *R*^2^ = 0.272, Suppl. Table 5) compared to IPD.

None of the 5 AR-PD patients with decreased myocardial 18 F-DOPA uptake had previous or recent NMS. On the other hand, AR-PD patients who had constipation and RBD since the onset showed normal myocardial 18 F-DOPA uptake. There were no AR-PD patients who had hyposmia at the onset and the only 1 AR-PD patient who described hyposmia recently prior to imaging, had normal cardiac 18 F-DOPA uptake.

From the IPD patients who showed decreased myocardial 18 F-DOPA uptake, 6 had constipation (2 since the onset, 4 recently), 5 had RBD (3 since the beginning, 2 recently) and 7 had hyposmia (6 since the onset, 1 recently).

### Brain 18 F-FDG metabolism

The cortical 18 F-FDG uptake pattern was similar in AR-PD and IPD. Hypometabolism was more conspicuous in the mesial prefrontal, inferior parietal and temporal cortices. The least affected cortical areas were the primary visual and insular cortex. No hypometabolism was observed in the posterior cingulate and precuneus. In subcortical regions, the number of patients with hypometabolism in the caudate, putamen and thalamus was relatively higher in AR-PD compared to IPD (Table 3).

**Table 3:** Brain 18 F-FDG PET/CT findings in subcortical regions

	AR-PD *n* (%)			IPD *n* (%)			*p*
	Hypermet.^a^	Normal	Hypomet.^b^	Hypermet.^a^	Normal	Hypomet.^b^	
Caudate	–	9 (47)	10 (53)	1 (6)	11 (65)	5 (30)	0.045*
Putamen	2 (10)	10 (53)	7 (37)	3 (18)	13 (76)	1 (6)	0.070
Thalamus	1 (5)	8 (42)	10 (53)	2 (12)	10 (59)	5 (29)	0.096

*Bold value represents statistically significant result

^a^Hypermetabolism

^b^Hypometabolism

### Rs-fMRI and DTI

BGN maps showed activity of the caudate, putamen, globus pallidus, and thalamus in healthy controls (Suppl. Fig. 1a) and PD patients (Suppl. Fig. 1b, c). It was also observed that the anterior cingulate cortex (ACC) contributed to BGN in PD groups (Suppl. Fig. 1b, c). There was no significant functional activity alteration between PD groups.

The TBSS analysis (Suppl. Fig. 2) revealed that AR-PD had a significant decrease in FA in WM areas, such as limbic structures including mesial temporal, bilateral caudocinguli and fornices, extralimbic structures including internal and external capsules, superior longitudinal fasciculus compared to IPD. In addition, significantly reduced FA is observed in the cerebellum, brainstem, and superior cerebellar peduncles in AR-PD.

## Discussion

This study opens new perspectives using a multimodal imaging. There was a typical nigrostriatal neurodegeneration pattern in our IPD group, whereas in AR-PD, the caudate corpus was found to be the most affected region following the posterior putamen. Previous 18 F-DOPA studies have reported that AR-PD patients show similar neurodegeneration pattern as IPD [5, 21–23]. However, the caudate corpus was not evaluated in these studies. On the other hand, Scherfler et al. [23] reported that decreased 18 F-DOPA uptake in the caudate corpus in Parkin-positive PD patients like our result. The lateral fibers of the nigrostriatal system typically project to the putamen, whereas the medial fibers to the caudate. In IPD, the projections from the lateral part of the SN to the posterior putamen are affected earlier and greater extent than those to the caudate [6]. Our finding of a significant decreased 18 F-DOPA uptake in the caudate corpus in AR-PD is possibly due to more homogenous neuronal loss in SN and may indicate an imaging clue for this group.

Furthermore, Scherfler et al. showed significant 18 F-DOPA reductions in midbrain areas of Parkin-positive patients [23]. In another study, Parkin patients had more widespread reductions in nigral signal intensity compared to IPD [24]. In our study 18 F-DOPA uptake in SN was relatively decreased in AR-PD without reaching significance. This finding was supported by our TBSS finding of significant brainstem involvement. Consistent with the literature, 18 F-DOPA uptake in nuclei contralateral to the dominant side of the disease was more decreased in our IPD group. Conversely, the absence of this finding in the AR-PD suggests that the degeneration process is more symmetrical. Similar findings were reported previously [5, 21, 22]. However, this question of symmetry or asymmetry has to be taken with caution. Since the disease duration was longer in our AR-PD group, asymmetry may not be an expected finding at the advanced period. But, to our opinion, initially symmetric degeneration still remains a question mark.

There are a limited number of pilot studies evaluating cardiac innervation with 18 F-DOPA in IPD, showing reduced myocardial 18 F-DOPA uptake [19, 25]. We wanted to provide a preliminary evaluation of our patients by obtaining additional cardiac images while performing presynaptic dopaminergic imaging. To our knowledge, this study is the first semi-quantitative study to compare myocardial 18 F-DOPA uptake between AR-PD and IPD. Our preliminary results showed that IPD patients had significantly reduced myocardial 18 F-DOPA uptake, whereas the AR-PD group with greater reduction in striatal 18 F-DOPA uptake showed relatively preserved myocardial dopaminergic innervation. AR-PD patients with decreased myocardial 18 F-DOPA uptake had a longer median disease duration than IPD patients, suggesting that cardiac denervation may occur later in the AR-PD group. Thus, peripheral involvement may occur in these patients at some point during the disease process, and we attempted to evaluate this by obtaining additional cardiac images while performing brain 18 F-DOPA PET/CT scan.

Basically, in midbrain areas, 18 F-DOPA has been reported to be taken up not only by dopaminergic neurons but also by serotoninergic and noradrenergic neurons expressing dopa decarboxylase [26, 27]. Considering this, decreased 18 F-DOPA uptake in the myocardium may reflect a widespread dysfunction, rather than being limited to the dopaminergic system alone. If a patient exhibits decreased 18 F-DOPA uptake in the myocardium, cardiac denervation should also be considered. However, if 18 F-DOPA uptake is relatively preserved in the myocardium, cardiac denervation could likely be ruled out. In fact, larger studies comparing 18 F-DOPA with 123I-MIBG should be planned. However, the difficulty of repeated clinic visits for debilitated PD patients, the high cost of 123I-MIBG and small number of AR-PD patients limit such study design.

The term “brain-first” or “body-first” has been proposed in recent classifications of IPD. However, this concept is not yet well established in monogenic PD. Our study, due to its cross-sectional design, reveals the degree of striatal and myocardial 18 F-DOPA uptake in the PD patients during that time of period. Although we cannot claim that our results are definitive, they raised the possibility of a “brain-first” progression pattern in the AR-PD group and since there are some reports in the literature that this group may have a “brain-first” type progression based on clinical presentation [4], we thought so. To conclusively determine a “brain-first” progression, it is necessary to follow the patients with brain and heart imaging, maybe even with parasympathetic imaging, from the beginning of their diagnosis. Although it is difficult to reach a sufficient number of patients due to the relatively small number of AR-PD patients, we recommend planning a separate study with prospective follow-up.

The relationship between constipation and RBD with cardiac denervation in IPD has been shown previously [4]. Contrary to our expectation, AR-PD patients who had constipation and RBD did not show decreased myocardial 18 F-DOPA uptake. Reduced myocardial 18 F-DOPA uptake may be a separate indicator of peripheral a-syn pathology. These may be independent findings depending on the speed and impact degree of the disease. In our study, a lack of a pathological or molecular confirmation of peripheral a-syn pathology with a skin or salivary gland biopsy and cross-sectional design pose an important limitation for the confirmation of peripheral a-syn pathology and detection of the sequence of involvement.

Few studies have investigated brain 18 F-FDG PET imaging in monogenic PD [28, 29]. In our study, we observed a similar cortical 18 F-FDG uptake pattern between PD groups. However, in subcortical regions, the number of patients with hypometabolism in the striatum and thalamus was relatively higher in AR-PD group. In fact, the characteristic findings defined for PD are the presence of increased metabolism in the basal ganglia [8]. However, this is a finding in the early stages of disease. One study reported reduced striatal metabolism in advanced disease, in line with our result [30]. With disease progression, as the neuronal synaptic activity in the striatum decreases, a parallel progressive loss of striatal energy metabolism can be considered likely. Therefore, the number of AR-PD patients with hypometabolism in the striatum and thalamus was found to be higher may be explained by the longer disease duration. In addition, it is not clear whether the decrease in cortical metabolism is due to a-syn pathology or an innervation defect originating from the affected striatum. Considering that LP is not usually seen in patients with AR-PD, the presence of cortical hypometabolism in these patients is probably associated with an innervation defect.

Recent research has demonstrated the influence of Parkin gene polymorphisms on spontaneous brain functional activity at rest and WM microstructures [31, 32]. This study is the first to investigate resting-state functional activity of BGN and WM microstructures in AR-PD patients compared to those with IPD. We found no significant difference in functional BGN activity between PD groups. Both PD groups showed increased activity in ACC. Overactivation of the ACC was previously demonstrated in PD patients compared to controls [33]. The authors suggested that this overactivation might result from a recruitment mechanism aimed at compensating for the movement difficulties. In addition, we observed notable alterations in WM microstructures related to movement and cognition, marked by a corresponding decrease in FA in AR-PD patients compared to IPD. This suggests more extensive WM microstructural damage in AR-PD, consistent with the findings of Yu et al., who reported that PD patients with the Parkin gene polymorphisms exhibited more severe WM microstructural damage [34].

## Limitations

This multimodal imaging study provides valuable insights into the pathophysiology of AR-PD, but it has some limitations. While disease duration was longer in the AR-PD group, which may have influenced some findings, the validity of our striatum 18 F-DOPA results in both AR-PD and IPD patients with similar disease durations is reassuring, as shown in Fig. 1. In addition, multivariable regression analysis showed that it had no effect as a confounding factor. We also acknowledge the possibility of “brain-first” and “body-first” subtypes within the IPD group; but for this study, we focused specifically on AR-PD patients. Due to the difficulty of recruiting debilitated PD patients for repeated clinic visits, we were unable to obtain cardiac 123I-MIBG imaging for comparison with the cardiac 18 F-DOPA images. Our evaluation of BGN activity using rs-fMRI was limited, and we must interpret the comparison of DTI data between groups with caution due to the significant difference in disease duration. Nevertheless, we believe that this study offers important contributions to our understanding of AR-PD’s pathophysiology, paving the way for future research in this area.

## Conclusions

Compared to IPD, AR-PD patients showed an additional decrease in 18 F-DOPA uptake in the caudate corpus and displayed a more symmetrical denervation pattern, suggesting more severe and widespread loss of dopaminergic neurons. Our preliminary finding is that myocardial 18 F-DOPA uptake in AR-PD was similar to controls. 18 F-DOPA may be used as a practical tool for evaluating the dopaminergic innervation of brain and heart together. Acquiring additional cardiac imaging during brain 18 F-DOPA PET scan may be helpful for evaluating the pathophysiological process in the PD patients as a whole, but further evaluation is needed in this area. This reduces both the radiation exposure and costs. Regardless of the etiology, both groups had cortical hypometabolism. The presence of more severe striatal denervation, changes in cortical and subcortical 18 F-FDG metabolism and WM microstructure together with relatively preserved myocardial innervation raise the possibility of “brain-first” progression in AR-PD, and we recommend planning a separate study with prospective follow-up. Our results also suggest that although AR-PD and IPD are considered to have distinct pathogenetic mechanisms especially with regard to a-syn pathology, they may involve intersecting cellular pathways independent from a-syn leading to a similar sequence of disease process.

## Supplementary Information

Below is the link to the electronic supplementary material.

Supplementary file1 (TIFF 320 KB)

Supplementary file2 (TIFF 402 KB)

Supplementary file3 (DOCX 13 KB)

Supplementary file4 (PDF 119 KB)

Supplementary file5 (PDF 81 KB)

Supplementary file6 (PDF 83 KB)

Supplementary file7 (PDF 30 KB)

Supplementary file8 (PDF 30 KB)

## Data Availability

The datasets generated and/or analyzed during the current study are available from the corresponding author on reasonable request.
