# Supplementary figures and images for: Multimodal imaging analysis of autosomal recessive Parkinson’s disease
Source: Ann Nucl Med. 2025 Apr 24;39(8):813–22. doi: 10.1007/s12149-025-02053-4 (PMC12289758; doi:10.1007/s12149-025-02053-4)

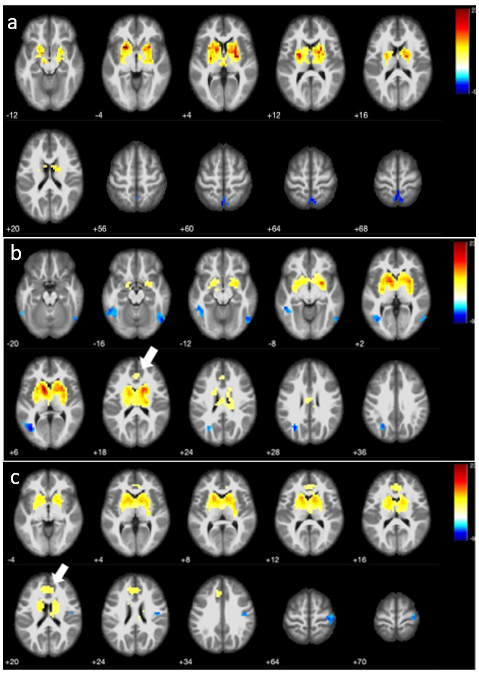

Supplement: Supplementary file 1 — Supplementary file1 (TIFF 320 KB) [file 12149_2025_2053_MOESM1_ESM.tiff]

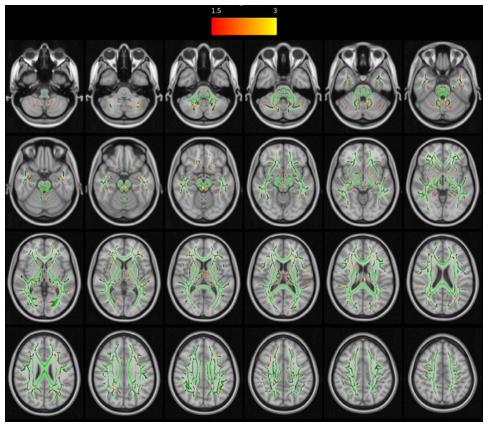

Supplement: Supplementary file 2 — Supplementary file2 (TIFF 402 KB) [file 12149_2025_2053_MOESM2_ESM.tiff]
