# Supplementary material for: Multimodal imaging analysis of autosomal recessive Parkinson’s disease
Source: Ann Nucl Med. 2025 Apr 24;39(8):813–22. doi: 10.1007/s12149-025-02053-4 (PMC12289758; doi:10.1007/s12149-025-02053-4)
Supplement: Supplementary file 3 — Supplementary file3 (DOCX 13 KB) [file 12149_2025_2053_MOESM3_ESM.docx]

**SUPPL. FIGURE LEGENDS**

**Suppl. Fig. 1** Resting-state basal ganglia network activity in healthy controls (a, n = 9), in AR-PD group (b, n = 17) and in IPD group (c, n = 15) [Color bar from red to blue indicates increased and decreased connectivity, respectively]. The contribution of anterior cingulate cortex to basal ganglia network in AR-PD and IPD shown by white arrow

**Suppl. Fig. 2** TBSS-DTI map reveals reduced FA in AR-PD when compared to IPD (significant voxels of red-to-yellow overlaid on the mean FA skeleton [green]; FWE-p<0.05)
