# Supplementary material for: Multimodal imaging analysis of autosomal recessive Parkinson’s disease
Source: Ann Nucl Med. 2025 Apr 24;39(8):813–22. doi: 10.1007/s12149-025-02053-4 (PMC12289758; doi:10.1007/s12149-025-02053-4)
Supplement: Supplementary file 4 — Supplementary file4 (PDF 119 KB) [file 12149_2025_2053_MOESM4_ESM.pdf]

Suppl. Table 1.

Patients' clinical characteristics and cognitive profiles

		AR-PD (n: 20)			IPD (n: 20)		p		Controls for 18F-DOPA PET		Controls for MRG (n: 9)
									Brain (n: 13)	Myocardium (n: 10)	
**Age** (year)		43.65 ± 9.09			52.25 ± 8.13		**0.003***		48,77 ± 11,05	48,60 ± 10,09	43,78 ± 6,64
**Age at disease onset** (year)		29.05 ± 8.48			46.20 ± 8.24		**<0.001***				
**Disease duration** (year)		13.50 (5-28)			5 (3-14)		**<0.001***				
**Hoehn-Yahr stage**		2 (1-3)			1 (1-3)		**<0.001***				
**UPDRS-III score**		16.50 (5-38)			12 (6-28)		0.074				
		**n (%)**			**n (%)**				**n (%)**	**n (%)**	**n (%)**
**Female**		11 (55)			9 (45)		0.527		7 (53.8)	7 (70)	5 (55.5)
**Lateralization (left/right)**		10 (50) / 10 (50)			11 (55) / 9 (45)		0.758				
**Non-motor symptoms**		Onset	Recent		Onset	Recent					
Constipation		6 (30)	6 (30)		2 (10)	9 (45)	0.114	0.327			
RBD		3 (15)	3 (15)		6 (30)	6 (30)	0.256	0.256			
Hyposmia		-	1 (5)		8 (40)	11 (55)	**0.002***	**0.001***			
	**n**			**n**							
**Education** (year)	20	11 (5 – 18)		16	14 (5 – 20)		0.150				
**Mini-mental state**	20	28 (18 – 30)		16	29 (21 – 30)		0.142				
**Memory**[a]											
Enhanced cued recall test	8	48 (42-48)		10	48 (45-48)		0.179				
**Executive functions**											
Trail making test B time (s)	20	137 (56 – 240)		15	115 (65 – 240)		0.546				
Trail making test B (Z-score)	20	1.80 (-1.18 – 6.5)		15	0.43 (-1.81 – 8.58)		0.309				
Stroop first part time (s)	20	10 (8 – 27)		15	9 (6 – 60)		0.056				
Stroop second part time (s)	20	12 (8 – 27)		15	11 (6 – 60)		0.074				
Stroop third part time (s)	20	14 (11 – 60)		15	13 (10 – 60)		0.153				
Stroop forth part time (s)	20	19 (13 – 60)		15	16 (12 – 90)		0.349				
Stroop fifth part time (s)	20	27 (19 – 90)		15	28 (17 – 90)		0.867				
**Attention**											
Trail making test A time (s)	20	45 (19-180)		16	38 (25-81)		0.293				
Trail making test A (Z-score)	20	0.36 (-2.77 – 12.11)		16	-0.81 (-2.67 – 2.10)		**0.048***				
**Language**											
Semantic fluency	20	15 (11 – 26)		16	20 (11 – 34)		0.069				
Semantic fluency (Z-score)	20	-0.58 ± 0.85		16	-0.17 ± 1.20		0.244				
Phonemic fluency	20	11.40 ± 4.84		16	12.63 ± 4.53		0.444				
Phonemic fluency (Z-score)	20	0.04 ± 0.86		16	0.02 ± 0.90		0.946				
**Visuospatial function**											
Clock drawing test	20	4 (1 – 4)		16	4 (3 – 4)		0.128				
**Beck depression score**	20	12 (2 – 31)		16	8 (4 – 43)		0.513				

[a]Memory performance of the remaining was evaluated with verbal memory processes test and all patients received a complete score.
UPDRS-III: Movement Disorder Society-Unified Parkinson's Disease Rating Scale Part III score, RBD: REM Behavioural Disorder
*Bold values represent statistically significant results.
