# Supplementary material for: Multimodal imaging analysis of autosomal recessive Parkinson’s disease
Source: Ann Nucl Med. 2025 Apr 24;39(8):813–22. doi: 10.1007/s12149-025-02053-4 (PMC12289758; doi:10.1007/s12149-025-02053-4)
Supplement: Supplementary file 5 — Supplementary file5 (PDF 81 KB) [file 12149_2025_2053_MOESM5_ESM.pdf]

Suppl. Table 2

Clinical features of PD groups with reduced or preserved myocardial 18F-DOPA uptake

	AR-PD (n = 19)			IPD (n = 20)		
	**Reduced**	**Preserved**	**p**	**Reduced**	**Preserved**	**p**
Age	43.20 ± 9.17	43.57 ± 9.70	0.942	52.08 ± 9.66	52.50 ± 5.70	0.914
Disease duration	13 (10-19)	13 (5-28)	0.950	5 (3-14)	5 (4-6)	0.750
Hoehn-Yahr stage	2 (2-2)	2 (1-3)	0.572	1 (1-3)	1 (1-3)	0.852
UPDRS-III score	16.40 ± 5.68	17.71 ± 9.99	0.786	12 (7-22)	11 (6-28)	0.462
