# Supplementary material for: Multimodal imaging analysis of autosomal recessive Parkinson’s disease
Source: Ann Nucl Med. 2025 Apr 24;39(8):813–22. doi: 10.1007/s12149-025-02053-4 (PMC12289758; doi:10.1007/s12149-025-02053-4)
Supplement: Supplementary file 6 — Supplementary file6 (PDF 83 KB) [file 12149_2025_2053_MOESM6_ESM.pdf]

Suppl. Table 3

Striatal and myocardial 18F-DOPA uptake results in DJ-1 patients

	**Specific region-to-occipital ratios**	**Ipsilateral**	**Contralateral**	**p**
Caudate head	1.89 ± 1.11	1.61 ± 0.77	2.10 ± 1.39	0.345
Caudate corpus	0.95 ± 0.44	0.98 ± 0.48	0.87 ± 0.36	0.397
Anterior putamen	1.84 ± 1.06	1.90 ± 1.10	1.34 ± 0.49	0.330
Posterior putamen	0.78 ± 0.32	0.71 ± 0.26	0.67 ± 0.23	0.340
Substantia nigra	0.64 ± 0.41	0.36 ± 0.38	0.45 ± 0.32	0.664
	**DJ-1 (n = 3)**	**Controls (n = 10)**		
Septum/Mediastinum	1.93 ± 0.92	1.83 ± 0.30		0.584
Lateral wall/Mediastinum	1.95 ± 0.22	1.62 ± 0.16		0.015
Apex/Mediastinum	1.63 ± 0.12	1.54 ± 0.26		0.584
Whole-myocardium /Mediastinum	1.84 ± 0.10	1.66 ± 0.23		0.247
