# Supplementary material for: Multimodal imaging analysis of autosomal recessive Parkinson’s disease
Source: Ann Nucl Med. 2025 Apr 24;39(8):813–22. doi: 10.1007/s12149-025-02053-4 (PMC12289758; doi:10.1007/s12149-025-02053-4)
Supplement: Supplementary file 7 — Supplementary file7 (PDF 30 KB) [file 12149_2025_2053_MOESM7_ESM.pdf]

Suppl. Table 4

Multivariable linear regression analysis for 18F-DOPA uptake in caudate corpus.

	**Unstandardized Coefficients**		**95% Confidence Interval**		**Standardized Coefficients**	**p**
	**B**	**Standard Error**	**Lower Limit**	**Upper Limit**		
AR-PD	-0.624	0.158	-0.944	-0.304	-0.539	**<0.001***

*Bold value represents statistically significant results.
