# Supplementary material for: Multimodal imaging analysis of autosomal recessive Parkinson’s disease
Source: Ann Nucl Med. 2025 Apr 24;39(8):813–22. doi: 10.1007/s12149-025-02053-4 (PMC12289758; doi:10.1007/s12149-025-02053-4)
Supplement: Supplementary file 8 — Supplementary file8 (PDF 30 KB) [file 12149_2025_2053_MOESM8_ESM.pdf]

**Suppl. Table 5**

Multivariable linear regression analysis for myocardial 18F-DOPA uptake.

| | Unstandardized Coefficients | | 95% Confidence Interval | | Standardized Coefficients | p |
|---|---|---|---|---|---|---|
| | **B** | **Standard Error** | **Lower Limit** | **Upper Limit** | | |
| AR-PD | 0.236 | 0.072 | 0.090 | 0.383 | 0.473 | **0.002*** |

*Bold value represents statistically significant results.
